# The Efficacy, Safety, and Side Effects of Intrapericardial Triamcinolone Treatment in Children with Post-surgical Pericardial Effusion: A Case Series

**DOI:** 10.1007/s00246-021-02704-z

**Published:** 2021-08-17

**Authors:** Manon H. van der Werff, Hetty J. van der Kamp, Johannes M. P. J. Breur

**Affiliations:** 1 Department of Pediatric Cardiology, University Medical Center, PO Box 85090, 3508 AB Utrecht, The Netherlands; 2 Department of Pediatric Endocrinology, University Medical Center, PO Box 85090, 3508 AB Utrecht, The Netherlands

**Keywords:** Pericardial effusion, Intrapericardial, Triamcinolone, Adrenal insufficiency

## Abstract

Intrapericardial triamcinolone can be used to treat chronic pericardial effusion (PE) in adults; however, pediatric data are lacking. In this case series we aim to evaluate the efficacy, safety, and side effects of intrapericardial triamcinolone in children with PE. The incidence and treatment of post-surgical PE from 2009 to 2019 were determined using the institutional surgical database and electronic patient records. Furthermore, a retrospective analysis of efficacy, safety, and side effects of intrapericardial triamcinolone treatment for chronic post-surgical PE was performed. The incidence of postoperative PE requiring treatment was highest after atrial septal defect (ASD) closure when compared to other types of cardiac surgery (9.7% vs 4.3%). Intrapericardial treatment with triamcinolone resolved pericardial effusion in 3 out of 4 patients. All patients developed significant systemic side effects. Surgical ASD closure is associated with an increased risk of development of PE requiring treatment. Intrapericardial triamcinolone is an effective treatment for chronic postoperative PE in children, but is always associated with significant systemic side effects. Close monitoring and treatment of adrenal insufficiency are mandatory in these cases.

**Supplementary Information:**

The online version contains supplementary material available at 10.1007/s00246-021-02704-z

## Introduction

The reported prevalence of postoperative pericardial effusion (PE) ranges between 1 and 53% [1–8] in children and adults. PE is often responsive to anti-inflammatory therapy, but in a minority of the cases PE becomes chronic. Possible treatment options for chronic PE are pericardiocentesis, creation of a surgical pericardial window, and intrapericardial triamcinolone. Intrapericardial triamcinolone has been associated with dramatic symptomatic improvement in adults [9]. There are no recommendations on intrapericardial triamcinolone treatment of PE in children. It is only known that corticosteroid use should be restricted because of the side effects [10]. The aim of this study was to establish the incidence of PE requiring treatment after pediatric cardiac surgery and to evaluate the efficacy, safety, and side effects of intrapericardial triamcinolone treatment in chronic postoperative pediatric PE.

## Methods

### Study Population

The institutional surgical database was used to identify all pediatric patients who received cardiac surgery for congenital heart disease (CHD) in 2019 and all surgical atrial septal defect (ASD) closures from 2009 to 2019. Electronic patient records and echo database were checked for postoperative occurrence and treatment of PE. PE treatment was initiated when echocardiography showed hemodynamically significant PE with or without clinical signs of cardiac tamponade.

Furthermore all patients receiving intrapericardial triamcinolone treatment between 2014 and July 2020 were identified using the hospital database. Medical records, echocardiography reports, laboratory markers, medication, and operation reports from before and after the treatment with triamcinolone were reviewed.

### Intervention

Intrapericardial triamcinolone was administered to patients with postoperative chronic (> 3 months) and/or recurrent PE, despite other treatments. Installation of triamcinolone (300 mg/m^2^—according to *Maisch *et al*.* [9]) was performed using a pigtail drain after pericardiocentesis. After 24 h the remaining pericardial fluid was aspirated and the drain was removed.

### Outcome

The outcome measures were efficacy of PE treatment, the presence of side effects, and the adrenal function. The efficacy of triamcinolone was measured by the persistence or disappearance of PE on echocardiograms performed after treatment. The presence of side effects was extracted from the medical records. The adrenal function was determined by low-dose (1mcg) ACTH-tests.

## Results

All patients who developed significant post-surgical PE were primarily treated with prednisone. In the CHD group (ASD surgery excluded) 4.3% (7/164) required PE treatment versus 9.7% (28/289) in the ASD group. Prednisone treatment alone was effective in all patients in the CHD group (7/7) and in 82% (23/28) of the ASD group. Recovery occurred after a mean of 8 days (range 4–16) in the CHD group and after 7 days (range 4–27) in the ASD group. Pericardiocentesis was performed in 1.7% (5/289 patients) of the ASD group.

Four patients were treated with intrapericardial triamcinolone treatment. Patient characteristics and outcome are summarized in Table 1. Three of these patients underwent surgical ASD closure and one patient underwent epicardial pacemaker implantation. The time between onset of PE and triamcinolone administration varied between 1.5 and 9 months. All patients were treated with prednisone and pericardiocentesis (1–4 times) before triamcinolone administration. One patient was treated with intravenous immunoglobulins (IVIG) [11]. In three out of four patients PE did not reoccur after triamcinolone administration. In one patient PE reoccurred and a surgical pericardial window was created.

**Table 1:** Patient characteristics and the efficacy of triamcinolone

No	Age (years) / sex	Operation	Time between onset PE and triamcinolone administration	Number of times pericardiocentesis before triamcinolone administration	Number of times prednisone was started or dose was increased before triamcinolone administration	Number of times treated with IVIG before triamcinolone administration	Developed reoccurrence PE after triamcinolone administration
1	11/F	ASD surgical closure	9 months	1	4	2	No
2	11/M	Pacemaker implantation	2.5 months	1	2	0	No
3	1/F	ASD surgical closure	7 months	4	2	0	No
4	14/M	ASD surgical closure	1.5 months	3	2	0	Yes

*PE* pericardial effusion, *IVIG* intravenous immunoglobulin, *ASD closure* atrial septal defect closure

The course of the side effects of triamcinolone treatment is summarized in Table 2. All patients developed symptoms within 3 weeks lasting up to several years.

**Table 2:** Specifics of the symptoms after triamcinolone administration

No	Time between triamcinolone administration and start symptoms	Type of symptoms	Duration of symptoms	Time between triamcinolone administration and adequate ACTH-test
1	1 week	Fatigue, low energy levels	Not clear	1 year and 9 months
2	1 week	Fatigue, easily tired, irritable, quickly bad-tempered	3 years and 5 months^a^	Not adequate yet
3	1 day	Fatigue, low energy levels, whiny, bad-tempered, mood swings, restless	4 months^a^	Not performed yet
4	3 weeks	Fatigue, low energy levels, decreased fitness, difficulty falling asleep	3 years and 7 months	3 years and 7 months

^a^Still ongoing

*ACTH-test* adrenocorticotropic hormone test

### Case Reports

#### Patient 1

A 10-year-old girl with multiple congenital dysmorphisms and a spontaneously closed ventricular septal defect underwent surgical ASD closure. PE developed 1 month later and prednisone was started. In a period of 9 months prednisone was unsuccessfully tapered 4 times. Two months after prednisone initiation a Cushingoid face developed and after 5 months she developed fatigue (low energy levels, early bedtimes, and missing school). Additional treatment consisted of pericardiocentesis IVIG (twice) and colchicine [10], all without success. After 9 months of persisting PE pericardiocentesis was performed with administration of 420 mg triamcinolone and 80 mg gentamycin (sclerotherapy). Three days after triamcinolone administration, the morning cortisol concentration was < 0.02 µmol/L. Thereafter PE did not reoccur. However she developed extreme fatigue. Hydrocortisone supplementation (12 mg/m2) was added for 8 months because of those signs and symptoms. Five months after hydrocortisone initiation a small improvement in fitness was seen, but it took one year and 9 months (after triamcinolone administration) until the ACTH-test normalized.

#### Patient 2

An 11-year-old boy underwent epicardial pacemaker implantation for paroxysmal atrioventricular block. PE occurred 1 week later and prednisone was started. Because of progressive PE and echocardiographic signs of tamponade pericardiocentesis was performed. Two attempts were made to taper prednisone without success. Two months after the start of prednisone a Cushingoid face developed. After 2.5 months a second pericardiocentesis was performed with administration of 420 mg triamcinolone. On the same day colchicine was started and prednisone was continued. There has been no recurrence of PE after the triamcinolone administration. One week after triamcinolone administration he developed symptoms of fatigue, expressed by being easily tired, irritable, and bad-tempered. Prednisone and colchicine were tapered within 2 and 3.5 months, respectively. Because of fatigue symptoms, expressed by low energy levels, missing school, and not having fun, prednisone was restarted 9 months later and the dose increased considerably in the following months. His listless feeling and increased need of sleep persisted despite a “physiologic” morning dose of prednisone. No other explanation for the fatigue could be established. Symptoms persist to date and ACTH-tests remain inadequate.

#### Patient 3

A 5-month-old girl underwent surgical ASD closure. PE with tamponade occurred one month later for which immediate pericardiocentesis was performed and prednisone was started. In a period of 7 months two attempts to taper prednisone were unsuccessful despite the addition of colchicine in the 6th month. Furthermore pericardiocentesis had to be performed twice. In the 6th month fatigue symptoms started, expressed by being easily tired, bad-tempered, and whiny. After the 7th month 140 mg triamcinolone was administered intrapericardially without recurrence of PE thereafter. One day after triamcinolone treatment fatigue symptoms started, expressed by being bad-tempered, listless, whiny, and having low energy levels. Three days after triamcinolone administration, the morning cortisol concentration was < 0.02 µmol/L. Because of these symptoms hydrocortisone (12 mg/m^2^) was started. Symptoms like being restless, mood swings, being bad-tempered, and sleeping badly continued for 2 months. Because of the suspicion of adrenal insufficiency, hydrocortisone was tapered. Up till now, the symptoms persist and hydrocortisone therapy is still necessary.

#### Patient 4

A 14-year-old boy with a history of surgically corrected esophageal atresia underwent surgical ASD closure. Two days later cardiac tamponade developed and acute pericardiocentesis was performed. After 1 week a second cardiac tamponade developed and another pericardial drainage was performed. Prednisone, NSAID, and colchicine were started. Three weeks after initiation of prednisone, a Cushingoid face was developed. One attempt was made to taper prednisone, which resulted in a relapse of PE and another pericardiocentesis. One and a half month after the onset of PE, pericardiocentesis was performed and 320 mg triamcinolone was administered intrapericardially. Nevertheless PE reoccurred and 1 week later a surgical pericardial window was created with continuation of prednisone treatment without reoccurrence of PE. One week later fatigue symptoms started, expressed by low energy levels, decreased fitness, and difficulty falling asleep causing tiredness during the day. As a result this patient was only able to attend half days at school. Melatonin and rehabilitation were initiated to improve sleep and physical fitness. Three months after creation of the pericardial window, prednisone was completely tapered. However it took 3 years and 4 months to obtain a normal ACTH-test. During this period the fatigue symptoms were varyingly present, only improving at the time of the adequate ACTH-test.

## Discussion

The incidence of pericardial effusion requiring medical treatment is high after surgical ASD closure (9.7%). In the vast majority of cases oral anti-inflammatory therapy led to complete resolution of PE. Chronic PE developed in 10% of patients after surgical ASD closure. The intrapericardial administration of triamcinolone resolved chronic PE in 3 out of 4 patients. However all patients had long lasting serious side effects.

The incidence of PE (requiring at least prednisone) after ASD closure was more than twice as high as the incidence after all cardiac surgeries (9.7% versus 4.3%). These findings are in line with other reports identifying ASD closure as an independent risk factor for readmission with PE and intervention [12]. It is assumed that chronic volume overload of the right atrium may support altered mechanics of PE production, combined with the inflammatory reaction secondary to the pericardiotomy and right atriotomy.

To the best of our knowledge, this is the first study evaluating the efficacy of intrapericardial triamcinolone in children. *Maisch *et al*.* [9] treated 84 adult patients with intrapericardial triamcinolone—54 patients with 600 mg/m^2^ and 30 patients with 300 mg/m^2^—preventing PE recurrence in 92.6% vs. 86.7% of patients after 3 months and in 86.0% vs 82.1% after 1 year. In this study intrapericardial triamcinolone (300 mg/m^2^) was immediately effective in three of four patients with chronic postoperative and/or recurrent PE. It is debatable whether or not a higher triamcinolone dose could have changed the course of the unresponsive patient. A lower dose than 300 mg/m^2^ was never considered, as those patients did not respond to already high doses of prednisone. However, the results suggest that a lower initial dose may be considered in a dose–response study.

All patients treated with intrapericardial triamcinolone developed side effects shortly after administration including fatigue, low energy levels, being easily tired, and bad-tempered. Patient 2 and 4 newly developed these symptoms after the administration and patient 1 and 3 had worsening of these symptoms not otherwise explained. All patients had good systolic and diastolic function, therefore a cardiac cause of the side effects is unlikely. The side effects lasted at least 2 years (excluding patient 3), much longer than the known duration of side effects for oral corticosteroids [12].

Although it may be assumed that intrapericardial administration of corticosteroids avoids major side effects as seen in systemic treatment, iatrogenic Cushing’s syndrome was described earlier by Grubb et al. [13]. It may be recommended to monitor the morning plasma cortisol shortly after treatment to confirm adrenal suppression. However, this is only a warning sign, as it takes 14–21 days to develop adrenal insufficiency. A concentration > 0.5 µmol/L (depending on the assay used) excludes triamcinolone absorption and systemic side effects.

*Maisch *et al*.* [9] report that iatrogenic Cushing syndrome developed in 13.3% of adult patients receiving 300 mg/m^2^ intrapericardial triamcinolone, without specifying their symptoms. Known symptoms of Cushing syndrome in children are growth retardation, delayed puberty, obesity, hypertension, severe fatigue, mood swings, anxiety, irritability, and loss of emotional control [14]. The patients in our case study had all those systemic side effects.

Although triamcinolone has a 1.25 stronger anti-inflammatory effect than prednisone and a longer biologic half-life, there must be another explanation for the prolonged duration of adrenal insufficiency and the extreme side effects despite supplementation of a maintenance dose of hydrocortisone [15].

Intrapericardial treatment with agents like steroids, gentamycin or chemotherapy may be associated with the development constrictive pericarditis due to sclerosing properties of the medication [10]. However this complication has not been described for triamcinolone to date. Diastolic function was closely monitored and remained normal in all patients.

### Recommendation

Intrapericardial triamcinolone is of additional value in the treatment of chronic pediatric postoperative pericardial effusion. However adrenal insufficiency is likely to develop. Therefore treatment should always be performed in close collaboration with a pediatric endocrinologist.

## Limitations

The number of patients treated with intrapericardial triamcinolone administration is low. It is hard to draw definite conclusions on treatment efficacy with these low numbers. An international registration could help to develop optimal treatment for chronic postoperative PE.

## Conclusion

PE is more common after pediatric surgical ASD closure compared to other congenital heart surgeries. The majority of cases can be managed by anti-inflammatory treatment, such as prednisone. Chronic and/or recurrent post-surgical PE can successfully be treated with intrapericardial triamcinolone administration in the majority of cases, but is always associated with serious systemic side effects. Therefore, patients receiving intrapericardial triamcinolone therapy should be closely monitored for development and treatment of adrenal insufficiency.

## Supplementary Information

Below is the link to the electronic supplementary material.

Supplementary file1 (XLSX 23 KB)

## Data Availability

Data from Table S1 is available.
